# Robust myocardial T2 and T2* mapping at 3T

**DOI:** 10.1186/1532-429X-14-S1-P306

**Published:** 2012-02-01

**Authors:** Arshad Zaman, David M Higgins, Marc Kouwenhoven, Ananth Kidambi, John P Greenwood, Sven Plein

**Affiliations:** 1 Division of Medical Physics, University of Leeds, Leeds, UK; 2 Cardiology, Multidisciplinary Cardiovascular Research Centre & Leeds Institute of Genetics, Health and Therapeutics, Leeds, UK; 3 Philips Healthcare, Guildford, UK; 4 Philips Healthcare, Best, Netherlands

## Summary

Myocardial hemorrhage can be assessed implementing T2 and T2* mapping techniques, however robust myocardial T2 and T2* mapping is challenging at 3T. The goal of this study was to test T2 and T2* myocardial mapping techniques at 3T, with potential improvement in image quality on a system employing B1 shimming, with two methods of for B0 shimming.

## Background

Hemorrhage in the core of acute myocardial infarction (AMI) and area at risk appear to be markers of prognosis, enabling targeted therapy and potentially changing outcome. Myocardial T2 and T2* mapping have been used to detect such tissue changes at 1.5T. Such investigation at 3T is challenging due to additional susceptibility variation (e.g. inferolateral artefact at the heart-lung-liver interface).

We studied T2 and T2* myocardial mapping techniques at 3T on a system employing B1 shimming, with two methods of B0 shimming.

## Methods

Fifteen volunteers and three STEMI patients were scanned on a 3T Philips Achieva TX system with a 32-channel cardiac coil. Triggered, single breath-hold, multi-echo sequences were employed from which T2* and T2 maps were calculated. For the T2 map, the shot of 24 refocused spin echoes was subdivided into six groups, with a linear k-space order within each group contributing to a separate k-space. This strategy allows data acquisition within a breath hold. The echoes used for the centre of k-space for each image/group had consistent parity.

Conventional first-order volume B0 shimming (over a cuboid encompassing the whole heart and descending aorta, from which the on-resonance signal is maximised) was compared with image based (IB) B0 shimming, for which first- and second-order field adjustments are calculated from B0 maps, to maximise B0 map homogeneity over an arbitrarily-shaped volume prescribed by the user which can be drawn around the heart (ShimTool; Schär et al MRM 2010).

In all cases, septal, anterior and posterior ROIs were manually drawn to obtain average T2 or T2* values.

## Results

**Figure 1:**
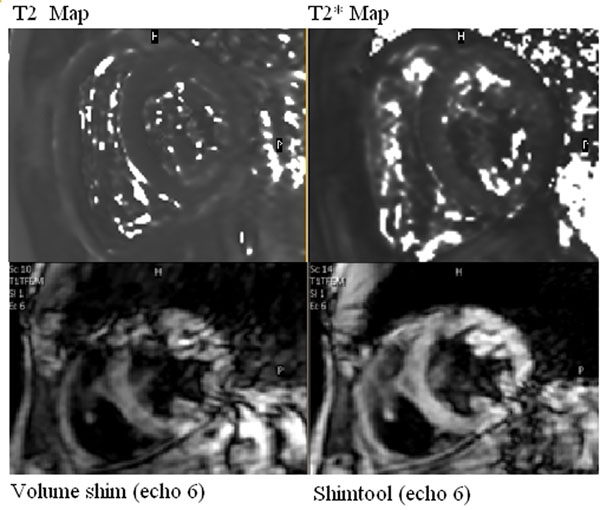
Top: T2 and T2* maps (healthy volunteer). Bottom: Sixth of six gradient echoes used in the T2* map calculation (TE=14ms). The septal signal is robust; lateral wall signal is sometimes improved with image-based B0 shimming (ShimTool).

Figure 1 shows typical T2 and T2* maps obtained in healthy volunteers. T2 and T2* values are reported in Table 1.

**Table 1:** Mean (StDev) of T2 and T2* ROIs (ms).

MAP	SEPTAL	ANTERIOR	POSTERIOR
T2* (volume B0 shim)	27.8(5.2)	28.4(5.8)	15.9(8.3)
T2* (image-based B0 shim)	25.7(5.4)	25.3(5.9)	18.7(4.6)
T2 (volume B0 shim)	39.3(5.9)	40.7(6.2)	37.4(5.9)
T2 (image-based B0 shim)	40.4(7.4)	38.9 (5.2)	38.7(5.6)

In 9 of the total of 15 volunteers, IB shimming reduced T2* map heterogeneity, particularly in the inferolateral wall. In the remaining 6 volunteers, conventional volume shim and IB shimming performed equally. For the T2 mapping, no difference in artefact power between conventional volume shim and IB B0 shimming was detected, although homogeneity improved away from susceptibility artefact.

The patient data reflected a similar pattern with additional increase in T2 and T2* values in the affected MI territory.

## Conclusions

T2 mapping is robust on a B1-shimmed 3T system, with the modified k-space filling strategy employed here. Septal T2* mapping was robust; outside the septum IB B0 shimming can improve T2* maps but inferolateral susceptibility effects remain problematic. Some caution in interpreting T2* values outside the septum has to be taken. At 3T, T2 mapping may prove more useful, particularly outside of the septum.

## Funding

S.P and J.P.G received an unrestricted educational research grant from Philips Healthcare.

